# Patterns of care and survival for adolescents and young adults with acute leukaemia – a population-based study

**DOI:** 10.1038/sj.bjc.6690104

**Published:** 1999-02

**Authors:** C A Stiller, S Benjamin, R A Cartwright, J V Clough, D W Gorst, M E Kroll, J R Y Ross, K Wheatley, J A Whittaker, P R A Taylor, S J Proctor

**Affiliations:** Childhood Cancer Research Group, University of Oxford, 57 Woodstock Road, Oxford, OX2 6HJ, UK; Leukaemia Research Fund Centre for Clinical Epidemiology, University of Leeds, 17 Springfield Mount, Leeds, LS2 9NG, UK; Countess of Chester Hospital, Liverpool Road, Chester, CH2 1UL, UK; Royal Lancaster Infirmary, Ashton Road, Lancaster, LA1 4RP, UK; Northampton General Hospital, Cliftonville, Northampton, NN1 5BD, UK; Clinical Trial Service Unit, Radcliffe Infirmary, Woodstock Road, Oxford, OX2 6HE, UK; University of Wales College of Medicine, Heath Park, Cardiff, CF4 4XN, UK; Royal Victoria Infirmary, Queen Victoria Road, Newcastle upon Tyne, NE1 4LP, UK

**Keywords:** acute leukaemia, survival rates, place of treatment, clinical trials

## Abstract

We report a population-based study of patterns of care and survival for people with acute leukaemia diagnosed at age 15–29 years during 1984–94 in regions of England and Wales covered by specialist leukaemia registries. There were 879 patients: 417 with acute lymphoblastic leukaemia (ALL) and 462 with acute myeloid leukaemia (AML). For ALL, actuarial survival rates were 43% at 5 years after diagnosis and 37% at 10 years. Survival improved significantly between 1984–88 and 1989–94 for those aged 15–19 at diagnosis. Patients entered in national clinical trials and those not entered had similar survival rates. Survival rates were similar at teaching and non-teaching hospitals and at hospitals treating different numbers of study patients per year. For AML, survival rates were 42% at 5 years after diagnosis and 39% at 10 years. Survival improved significantly between 1984–88 and 1989–94. Patients entered in the Medical Research Council AML10 trial had a higher survival rate than those who were in the earlier AML9 trial. Survival did not vary with category of hospital. We conclude that survival has improved for adolescents and young adults with acute leukaemia but that there is at present no evidence that centralized treatment results in a survival benefit for patients in this age group. © 1999 Cancer Research Campaign


*British Journal of Cancer* (1999) **79**(3/4), 658–665
© 1999 Cancer Research Campaign
Article no. bjoc.1998.0104

### Patterns of care and survival for adolescents and young adults with acute leukaemia – a population-based study

**CA Stiller[1], S Benjamin[1], RA Cartwright[2], JV Clough[3], DW Gorst[4], ME Kroll[1], JRY Ross[5], K Wheatley[6], JA Whittaker[7], PRA Taylor[8] and SJ Proctor[8]**

[1]Childhood Cancer Research Group, University of Oxford, 57 Woodstock Road, Oxford OX2 6HJ; [2]Leukaemia Research Fund Centre for Clinical Epidemiology, University of Leeds, 17 Springfield Mount, Leeds LS2 9NG; [3]Countess of Chester Hospital, Liverpool Road, Chester CH2 1UL; [4]Royal Lancaster Infirmary, Ashton Road, Lancaster LA1 4RP; [5]Northampton General Hospital, Cliftonville, Northampton NN1 5BD; [6]Clinical Trial Service Unit, Radcliffe Infirmary, Woodstock Road, Oxford OX2 6HE; [7]University of Wales College of Medicine, Heath Park, Cardiff CF4 4XN; [8]Royal Victoria Infirmary, Queen Victoria Road, Newcastle upon Tyne NE1 4LP, UK

**Summary** We report a population-based study of patterns of care and survival for people with acute leukaemia diagnosed at age 15–29 years during 1984–94 in regions of England and Wales covered by specialist leukaemia registries. There were 879 patients: 417 with acute lymphoblastic leukaemia (ALL) and 462 with acute myeloid leukaemia (AML). For ALL, actuarial survival rates were 43% at 5 years after diagnosis and 37% at 10 years. Survival improved significantly between 1984–88 and 1989–94 for those aged 15–19 at diagnosis. Patients entered in national clinical trials and those not entered had similar survival rates. Survival rates were similar at teaching and non-teaching hospitals and at hospitals treating different numbers of study patients per year. For AML, survival rates were 42% at 5 years after diagnosis and 39% at 10 years. Survival improved significantly between 1984–88 and 1989–94. Patients entered in the Medical Research Council AML10 trial had a higher survival rate than those who were in the earlier AML9 trial. Survival did not vary with category of hospital. We conclude that survival has improved for adolescents and young adults with acute leukaemia but that there is at present no evidence that centralized treatment results in a survival benefit for patients in this age group.

**Keywords**: acute leukaemia; survival rates; place of treatment; clinical trials

Survival for children with acute leukaemia has improved dramatically; 3-year survival among children diagnosed in Britain during 1989–91 exceeded 80% for acute lymphoblastic leukaemia (ALL) and 50% for acute myeloid leukaemia (AML) (Stiller, 1994*a*). It is widely recognized that survival rates among adults are substantially lower (Proctor, 1994; Chessells et al, 1998). Recent population-based studies of adults relate to fairly small numbers of cases, however (Gorst and Johnson, 1992; Evensen et al, 1994; Proctor et al, 1995; Youngson et al, 1995), and none quoted results specifically for the age group immediately following childhood.

There is a continuing debate on the optimum pattern of organization of cancer services (Expert Advisory Group, 1995; Haward, 1995; Kerr et al, 1996; Taylor, 1996) and the possible effects of different patterns on survival (Stiller, 1994*b*; Selby et al, 1996). Recently, interest has focused on the particular needs of adolescents with cancer, and the first units specifically for the care of patients in this age group have recently been set up (Souhami et al, 1996). It therefore seems particularly appropriate to carry out a large population-based study of patterns of care and survival for adolescents and young adults with acute leukaemia.

### PATIENTS AND METHODS

Patients diagnosed with acute leukaemia at age 15–29 during 1984–94 were ascertained from the Leukaemia Research Fund Data Collection Study (DCS) (Cartwright et al, 1997), the leukaemia registers in the former Northern (Proctor et al, 1995), North-Western (Gorst and Johnson, 1992), Mersey and Oxford Regions of England and the Welsh Leukaemia Registry. There were 879 patients in the series, 417 with ALL and 462 with AML. As there are estimated to be 154 cases of acute leukaemia diagnosed at age 15–29 in England and Wales per year (Cartwright et al, 1997), we believe that this study includes half of all cases diagnosed during 1984–94. Table 1 shows the coverage by region and calendar period. Most of England and Wales was covered except for the south-east, East Anglia and West Midlands. There was some inter-regional variation in incidence, but the overall rates were similar to those found for the DCS, which has been found to be 98% complete for the 15–29 year age group (Cartwright et al, 1997), suggesting that the series is virtually complete for the population covered.

The hospital principally responsible for the care of each patient was determined from registry data. Hospitals were classified in two ways:

(1) by their mean annual number of new patients aged 15–29 with acute leukaemia during the study period;
(2) as teaching or non-teaching hospitals.

During the study period, three Medical Research Council (MRC) trials of treatment for ALL in patients aged 15 and over were open – UKALL IX (Durrant and Richards, 1993), UKALL XA (Durrant et al, 1997) and UKALL XII (Goldstone et al, 1997) – and two for AML – AML9 (Rees et al, 1996) and AML10 (Hann et al, 1997). The Clinical Trial Service Unit (CTSU) provided

*Received 16 March 1998*
*Revised 18 June 1998*
*Accepted 23 June 1998*

*Correspondence to:* CA Stiller

**Table 1** Regions of residence, years of diagnosis and diagnostic group for all patients in the study

| | | Number of patients (annual incidence per million) | | |
|---|---|---|---|---|
| **Region** | **Years** | **ALL** | **AML** | **Total** |
| Northern | 1984–94 | 60 (7.9) | 51 (6.8) | 112 (14.7) |
| Yorkshire | 1984–94 | 53 (5.8) | 59 (6.5) | 112 (12.3) |
| Trent | 1984–89 | 41 (6.2) | 63 (9.6) | 104 (15.8) |
| Wessex | 1985–94 (Dorset) 1988–94 (remainder) | 33 (6.6) | 30 (6.0) | 63 (12.6) |
| Oxford | 1988–94 | 31 (8.5) | 43 (11.8) | 74 (20.4) |
| South West | 1984–94 | 66 (8.4) | 66 (8.4) | 133 (16.8) |
| Mersey | 1987–94 | 23 (5.4) | 32 (7.5) | 55 (12.9) |
| North West | 1984–94 | 68 (6.8) | 76 (7.6) | 144 (14.5) |
| Wales | 1984–94[a] | 42 (7.5) | 42 (7.5) | 84 (14.9) |
| Total | | 417 (7.0) | 462 (7.8) | 879 (14.8) |

[a]Gwent, Glamorgan and Dyfed, 1984–89 and 1991–94; Clwyd, 1987–94; Gwynedd and Powys, 1991–94.

information on patients aged 15–29 who were included in these trials.

Identifying information on all patients was sent to the National Health Service Central Register (NHSCR) for tracing and flagging. The NHSCR sent copies of death certificates for traced patients who had died and also notified us of those who had emigrated with resulting loss to follow-up. The remaining flagged patients were assumed to be alive on 30 November 1997. Death certificates or follow-up information were available by these means for all but 18 (2.0%) of the study series. Follow-up information was also available from the regional leukaemia registers and CTSU, including for some patients who could not be traced at NHSCR. Only eight (0.9%) had no follow-up from any source.

Survival rates were calculated by standard actuarial methods. Differences between survival curves were tested by log-rank tests and the $\chi^2$ test for linear trend. Information on remission and relapse was not generally available. In addition to analysing survival from the date of diagnosis, therefore, we did two further sets of analyses:

(1) survival starting at 4 weeks after diagnosis for both ALL and AML, in order to exclude patients who had died early, before treatment could take effect;
(2) survival starting at 2 years after diagnosis for ALL and 1 year after diagnosis for AML, in order to study the subsequent survival of patients who survived long enough to receive a full course of treatment.

### RESULTS

### Treatment hospitals

Table 2 shows the numbers of cases of ALL and AML treated at different categories of hospital. Twenty-nine per cent of patients were from hospitals seeing on average less than one new case per year of acute leukaemia in this age group. Over half were treated at teaching hospitals. Twenty-five younger patients (2.8% of the whole series, 6.8% of those aged 15–19) were treated in paediatric units. The distributions by hospital category were very similar for ALL and AML.

### MRC trials

Overall, 38% of patients were entered in MRC trials (Table 3), and the proportions were very similar for ALL and AML. Among patients with ALL, the entry rate was higher for those aged 15–19 at diagnosis (92/222, 41%) than for those aged 20–29 (62/195, 32%), though the difference was not quite statistically significant ($\chi^2$ = 3.74 on 1 d.f., $P$ = 0.053); for AML, entry did not vary substantially with age. For ALL, patients at teaching hospitals were more likely to be entered in MRC trials ($\chi^2$ = 24.8 on 1 d.f., $P$ < 0.0005), as were those at hospitals treating at least one study patient per year ($\chi^2$ = 12.8 on 1 d.f., $P$ < 0.0005). For AML, the entry rate varied between categories of hospital much less than for ALL and any differences were non-significant. In the four regions contributing data for the entire study period, the entry rates to ALL trials were similar during 1984–88 and 1989–94, 34% and 31% respectively. For AML, however, entry rose from 20% to 36% between the two periods. Patients in the North-East ALL III trial (Proctor et al, 1985) and other regional or single-institution studies were counted as non-trial throughout.

**Table 2** Main treatment hospital for patients in the study

| | ALL | AML | Total |
|---|---|---|---|
| New cases per year in age group | | | |
| <1 | 127 (30%) | 124 (27%) | 251 (29%) |
| 1 | 82 (20%) | 89 (19%) | 171 (19%) |
| 2–3 | 79 (19%) | 112 (24%) | 191 (22%) |
| 4–5 | 122 (29%) | 132 (29%) | 254 (29%) |
| Hospitals outside study region | 5 (1%) | 3 (1%) | 8 (1%) |
| Teaching | 235 (56%) | 276 (60%) | 511 (58%) |
| Other | 180 (43%) | 184 (40%) | 364 (41%) |
| Unknown | 2 | 2 | 4 |
| Total | 417 | 462 | 879 |

© Cancer Research Campaign 1999

**Table 3** Entry to MRC leukaemia trials for patients in the study

| | ALL | AML | Total |
|---|---|---|---|
| New cases per year at hospital | | | |
| <1 | 31/127 (24%) | 43/124 (35%) | 74/251 (29%) |
| 1–5 | 123/283 (43%) | 137/333 (41%) | 260/616 (42%) |
| Teaching hospitals | 112/235 (48%) | 115/276 (42%) | 227/513 (44%) |
| Other hospitals | 42/180 (23%) | 65/184 (35%) | 107/364 (29%) |
| Total | 154/417 (37%) | 180/462 (39%) | 334/879 (38%) |

### ALL survival

The actuarial survival rates for all patients with ALL were 77% at 1 year, 60% at 2 years, 51% at 3 years, 43% at 5 years and 37% at 10 years. The results by sex, age, year of diagnosis, entry to MRC trials and place of treatment are summarized in Table 4. While males had a lower survival rate than females, the difference was not significant. There was a significant trend of worsening survival with increasing age at diagnosis. For patients aged 15–19, there was a particularly marked improvement between 1984–88 (5-year survival 42%) and 1989–94 (58%) ($P = 0.017$), whereas for those aged 20–24 and 25–29 the increases in survival (from 34% to 42% and from 33% to 36% respectively at 5 years) were much more modest and not statistically significant (Figure 1). Survival was very similar for MRC trials and non-MRC patients. There was no evidence of any difference in survival between teaching and non-teaching hospitals, or between hospitals treating different numbers of study patients per year. This finding applied equally to MRC and non-MRC patients. Mortality during the first 4 weeks after diagnosis was 5% (20/411); of these 20 early deaths, eight (40%) occurred during the first week. The main causes of death were sepsis, tumour lysis and bleeding. Early mortality did not vary significantly with sex, age, calendar period, entry in the MRC trials or hospital category. When deaths in the first 4 weeks were excluded, the trend of worse survival with increasing age was still significant and the improvement between 1984–88 and 1989–94 for patients aged 15–19 was more evident ($P = 0.007$).

Among patients known to have survived 2 years after diagnosis, the difference in subsequent survival between the sexes was slight, 73% of males surviving a further 3 years compared with 71% of females. The trend with age was also non-significant, though patients aged 15–19 had a somewhat higher chance of surviving a further 3 years (76%) than those aged 20–29 (67%). The improvement between 1984–88 and 1989–94 for patients aged 15–19 was

**Table 4** Results of survival analyses for ALL

| Factor | Five-year survival (%) (s.e.) | All patients | | | After 4 weeks | | | After 2 years | | |
|---|---|---|---|---|---|---|---|---|---|---|
| | | O | E | P | O | E | P | O | E | P |
| Sex | | | | | | | | | | |
| Male | 41 (3.0) | 172 | 159.6 | 0.10 | 160 | 146.4 | 0.057 | 53 | 52.6 | 0.94 |
| Female | 48 (4.3) | 76 | 88.4 | | 68 | 81.6 | | 31 | 31.4 | |
| Age (years) | | | | | | | | | | |
| 15–19 | 49 (3.4) | 122 | 141.4 | | 111 | 130.7 | | 44 | 50.1 | |
| 20–24 | 38 (4.6) | 76 | 66.8 | 0.014 | 73 | 61.2 | 0.017 | 27 | 21.9 | 0.29 |
| 25–29 | 34 (5.5) | 51 | 40.8 | | 45 | 37.1 | | 13 | 12.0 | |
| Year | | | | | | | | | | |
| 1984–88 | 39 (3.4) | 139 | 125.1 | 0.076 | 130 | 115.1 | 0.048 | 54 | 43.2 | 0.016 |
| 1989–94 | 49 (3.5) | 110 | 123.9 | | 99 | 113.9 | | 30 | 40.8 | |
| MRC trial | | | | | | | | | | |
| Yes | 44 (4.1) | 92 | 95.7 | 0.63 | 84 | 88.2 | 0.57 | 35 | 32.4 | 0.56 |
| No | 43 (3.1) | 157 | 153.3 | | 145 | 140.8 | | 49 | 51.6 | |
| Hospital type | | | | | | | | | | |
| Teaching | 44 (3.3) | 138 | 140.4 | 0.75 | 128 | 129.1 | 0.88 | 45 | 46.7 | 0.71 |
| Other | 43 (3.7) | 109 | 106.6 | | 99 | 97.9 | | 38 | 36.3 | |
| Study patients per year at hospital | | | | | | | | | | |
| <1 | 41 (4.4) | 78 | 70.0 | | 70 | 63.9 | | 20 | 23.8 | |
| 1 | 49 (5.6) | 44 | 54.6 | 0.61 | 42 | 50.5 | 0.88 | 19 | 19.4 | 0.30 |
| 2–3 | 43 (5.7) | 50 | 47.5 | | 45 | 43.6 | | 17 | 16.0 | |
| 4–5 | 42 (4.5) | 73 | 73.0 | | 68 | 67.0 | | 27 | 23.8 | |

O, observed number of deaths; E, expected number of deaths; P, two-sided P-value from log-rank test (sex, year, MRC trial and hospital type) or test for trend (age and study patients per year).

 © Cancer Research Campaign 1999

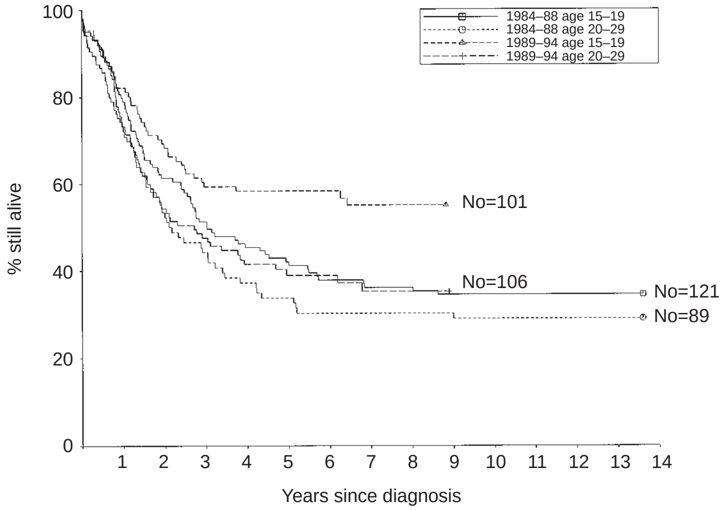

**Figure 1** Actuarial survival curves for study patients with ALL diagnosed 1984–88 and 1989–94

still highly significant, 69% and 85% respectively surviving a further 3 years ($P$ = 0.0094). Relapsed leukaemia accounted for 68/84 (81%) of deaths after 2 years, with 5/84 (6%) dying from complications of bone marrow transplantation. Deaths from relapsed leukaemia occurred throughout the follow-up period, the longest survivor dying 9 years after diagnosis.

### AML survival

Patients with AML had actuarial survival rates of 62% at 1 year, 48% at 2 years, 46% at 3 years, 42% at 5 years and 39% at 10 years. Thus, although the risk of death during the year following diagnosis was appreciably greater than for ALL, long-term survival for AML was slightly better. Other results are summarized in Table 5. As with ALL, survival was lower for males than for females, but the difference was non-significant. In contrast to ALL, however, there was little evidence of any variation with age at diagnosis. Survival improved significantly between 1984–88 and 1989–94 (Figure 2). During 1984–88, when the AML9 trial was open, there was hardly any difference in survival between MRC and non-MRC patients (35% and 37% at 5 years). During 1989–94, however, when patients were being entered in AML10, survival was significantly higher for trial patients than for those not entered (55% and 39% at 5 years, $P$ = 0.012). Thus survival in AML10 was substantially higher than in the AML9 trial, but there was little change between the two calendar periods for patients not in these trials. As with ALL, there was no evidence of variation in survival between hospital categories overall, or within the MRC and non-MRC groups.

Mortality in the first 4 weeks was 9% (43/460); 31 of these 43 deaths were in the first week. Early mortality did not vary significantly with sex, age or calendar period. Deaths within the first month occurred in 16/80 (20%) of patients in the M3 trial but only 27/380 (7%) of those with other subtypes of AML ($\chi^2$ = 11.5 on 1 d.f., $P$ = 0.0006). Eleven (79%) of the 14 M3 patients who died within 7 days had intracranial haemorrhage secondary to disseminated intravascular coagulation, compared with 4/17 (24%) for other subtypes. There was only one death of an M3 patient from intracranial haemorrhage after the first week. The other main cause of early death was sepsis. Most patients were diagnosed before October 1992, when all-*trans*-retinoic acid became available for treatment of M3 AML (Warrell et al, 1991). We found no significant decrease in early deaths from intracranial haemorrhage in M3 after that date, but the numbers were very small. Early mortality was lower at teaching than at non-teaching hospitals (5.4% vs 15%, $\chi^2$ = 10.3 on 1 d.f., $P$ = 0.0006) and lower at hospitals treating larger numbers of study patients, ranging from 3.8% at those with 4–5 per year to 14% at those with under one per year ($\chi^2$ = 7.53 on 1 d.f. for trend, $P$ = 0.0061). Patients entered in MRC trials were also less likely to die in the first 4 weeks (4.4%) than those who were not (12%; $\chi^2$ = 7.34 on 1 d.f., $P$ = 0.0067). Variations by sex and age in survival after 4 weeks remained non-significant, but the improvement between 1984–88 and 1989–94 was still significant, as was the difference in survival between MRC and non-MRC patients in 1989–94.

Among patients surviving at least 1 year, the subsequent survival rate did not vary significantly with sex or age at diagnosis. The improvement between 1984–88 and 1989–94 was also much diminished and non-significant, the chance of surviving a further 4 years increasing only from 65% to 69%. Survival after 1 year was significantly *worse* for MRC trial patients (56% surviving 4 more years) than for non-MRC patients (69%) diagnosed during 1984–88 ($P$ = 0.029), whereas for 1989–94 it was significantly better (77% MRC, 61% non-MRC; $P$ = 0.029). Relapsed leukaemia was the main cause of death beyond 1 year from diagnosis (72/104, 69%), with complications of bone marrow transplant accounting for 6/104 (6%) of deaths. The latest death from relapsed leukaemia was at 7 years 7 months.

### DISCUSSION

Over the past 25 years much attention has been given to the undeniably spectacular improvements in the survival of children with ALL and, latterly, AML. This study shows that progress in

© Cancer Research Campaign 1999

**Table 5** Results of survival analyses for AML

| Factor | Five-year survival (%) (s.e.) | All patients | | | After 4 weeks | | | After 1 year | | |
|---|---|---|---|---|---|---|---|---|---|---|
| | | O | E | P | O | E | P | O | E | P |
| Sex | | | | | | | | | | |
| Male | 38 (3.1) | 161 | 148.9 | 0.14 | 138 | 125.0 | 0.089 | 61 | 54.2 | 0.20 |
| Female | 46 (3.5) | 116 | 128.1 | | 96 | 109.0 | | 43 | 49.8 | |
| Age (years) | | | | | | | | | | |
| 15–19 | 42 (4.1) | 90 | 90.1 | | 80 | 76.3 | | 40 | 33.7 | |
| 20–24 | 41 (3.9) | 97 | 95.7 | 0.93 | 78 | 80.8 | 0.71 | 33 | 36.4 | 0.26 |
| 25–29 | 43 (4.0) | 90 | 91.2 | | 76 | 76.9 | | 31 | 33.9 | |
| Year | | | | | | | | | | |
| 1984–88 | 36 (3.4) | 135 | 116.0 | 0.020 | 113 | 97.5 | 0.038 | 48 | 43.9 | 0.40 |
| 1989–94 | 46 (3.1) | 142 | 161.0 | | 121 | 136.5 | | 56 | 60.1 | |
| MRC trial | | | | | | | | | | |
| Yes | 48 (3.7) | 99 | 115.5 | 0.043 | 91 | 98.1 | 0.34 | 42 | 43.8 | 0.73 |
| No | 38 (2.9) | 178 | 161.5 | | 143 | 135.9 | | 62 | 60.2 | |
| Hospital type | | | | | | | | | | |
| Teaching | 43 (3.0) | 164 | 171.4 | 0.35 | 149 | 145.8 | 0.67 | 67 | 64.0 | 0.55 |
| Other | 40 (3.6) | 112 | 104.6 | | 85 | 88.2 | | 37 | 40.0 | |
| Study patients per year at hospital | | | | | | | | | | |
| <1 | 42 (4.5) | 73 | 70.1 | | 56 | 59.5 | | 20 | 27.4 | |
| 1 | 46 (5.3) | 49 | 55.3 | 0.61 | 40 | 47.2 | 0.55 | 19 | 21.9 | 0.047 |
| 2–3 | 35 (4.5) | 75 | 61.0 | | 65 | 50.9 | | 25 | 21.0 | |
| 4–5 | 45 (4.3) | 77 | 87.6 | | 72 | 75.4 | | 40 | 33.7 | |

O, observed number of deaths; E, expected number of deaths; P, two-sided *P*-value from log-rank test (sex, year, MRC trial and hospital type) or test for trend (age and study patients per year).

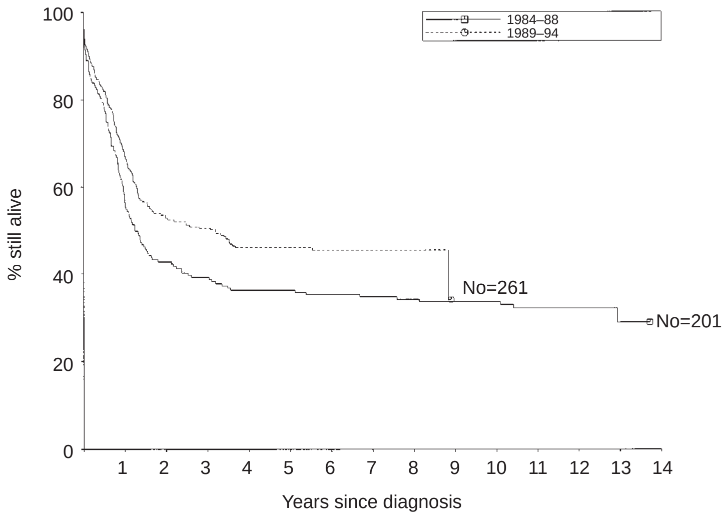

**Figure 2** Actuarial survival curves for study patients with AML diagnosed 1984–88 and 1989–94

the treatment of these diseases in adolescents and young adults has also led to substantial improvements in survival at the population level. Table 6 compares the changes in survival rates at age 15–29 with those found at age 0–14 over the same period in the National Registry of Childhood Tumours. For ALL, these results confirm on a population basis not only what has been found in the MRC trials, namely that age is an important indicator of prognosis beyond childhood (Chessells et al, 1998), but also that trends in survival among patients aged 20 and over have been disappointing. In the only other population-based study of trends in survival from leukaemia in adults (defined as people aged 15 and over, and presumably consisting predominantly of those aged over 30) no significant improvement was found between 1971–75 and 1986–90 (Badrinath et al, 1997). As the therapeutic approach in young adults has often been similar to that in children, this suggests that ALL in older patients differs biologically from ALL

 © Cancer Research Campaign 1999

**Table 6** Population-based 5-year survival rate (%) for acute leukaemia in Britain

| | Age at diagnosis (completed years) | | | | | | |
|---|---|---|---|---|---|---|---|
| | 0 | 1–4 | 5–9 | 10–14 | 15–19 | 20–24 | 25–29 |
| ALL | | | | | | | |
| 1984–88 | 26 | 81 | 72 | 55 | 42 | 34 | 33 |
| 1989–94 | 35 | 86 | 79 | 73 | 58 | 42 | 36 |
| AML | | | | | | | |
| 1984–88 | 23 | 35 | 40 | 37 | 40 | 37 | 30 |
| 1989–94 | 45 | 56 | 53 | 49 | 44 | 43 | 50 |

Sources: National Registry of Childhood Tumours (age 0–14); present study (age 15–29).

in childhood (Proctor, 1994). Survival of patients aged 16–21 who were treated on Children's Cancer Group protocols at paediatric oncology centres in North America was very similar to that for patients aged 10–15 (Nachman et al, 1993). In the UKALL XA trial, high hyperdiploidy, which is associated with a good prognosis, was more frequent among patients diagnosed aged 15–19 than among those aged 20–29, whereas the Philadelphia chromosome, which confers a poor prognosis, was less frequent (Secker-Walker et al, 1997). Taken together, these results indicate that further trials of treatment for childhood ALL might reasonably be applicable to most patients aged up to about 20 years, while a different approach is probably needed for the more resistant type of disease, which is increasingly common above that age. For AML, by contrast, there was little sign of systematic variation in prognosis with age and the survival rates were similar to those for children, suggesting that treatment approaches should be the same.

Population studies of patterns of care and survival should be interpreted cautiously because of the many possible confounding factors. Differences in prognostic factors between patients who are entered in trials and those who are not may well change over time. For example, a new trial using more aggressive treatment may recruit fewer low-risk patients while simultaneously excluding larger numbers of very ill patients because they are considered unfit for such treatment. Referral patterns may also vary according to perceived prognosis at presentation. Significant differences between groups of patients may of course arise by chance or result from incomplete control of confounding factors. Conversely, and especially when numbers are relatively small, real effects may be undetected. Nevertheless, such studies can provide valuable indications of the relative importance of standardized or centralized treatment for different cancers (Stiller, 1994*b*; Selby et al, 1996).

The only previous investigation of the effects of patterns of care on survival from leukaemia in adults covered lymphoid malignancy diagnosed at any age from 15 years upwards in the North-West Region of England during 1983–86 (Youngson et al, 1995). There was thus a slight overlap between that series and ours for ALL. Treatment according to a recognized protocol and treatment at the regional specialist oncology centre were each associated with significantly higher survival rates, largely because of the very poor survival of non-protocol patients outside the regional centre. The range of recognized protocols was not specified, however, and features of the single oncology centre other than its specialization may have been responsible for the higher survival rate observed there.

Children with ALL diagnosed in Britain during 1971–84 had a higher survival rate if they entered in the MRC trials *or* were treated at hospitals seeing larger numbers of children with this disease (Stiller and Draper, 1989). Among MRC trial patients, place of treatment made little difference to survival, and the only group identified as having a notably worse prognosis consisted of children who were not entered in the trials *and* were treated at a hospital with few patients. For childhood AML diagnosed during 1975–83, survival was worse among non-trial patients, but during 1984–88 [when children tended to be entered in the 'Joint AML' study (Marcus et al, 1987) rather than MRC trials] the difference had disappeared (Stiller and Eatock, 1994), as in the present study of young adults. In the earlier period children treated at teaching hospitals had a higher survival rate, but by 1984–88 too few children were treated at non-teaching hospitals for a meaningful comparison.

In the present study, patients with ALL had a similar survival rate whether or not they were entered in MRC trials. The high early mortality among non-MRC patients with AML presumably reflects a tendency not to enter severely ill patients in the trials, in some cases because they had died before they could be entered. Survival in AML10, which was open from 1988 to 1995, was better than in any previous randomized trial of treatment for AML (Hann et al, 1997). We have no information on the treatment given to individual patients, but the emergence since 1989 of a longer term survival advantage for patients who were entered in AML10 over those who were not is consistent with some more recently diagnosed non-trial patients receiving less effective treatment similar to that used during the previous 5 years.

In general, we found no survival advantage associated with treatment at hospitals with larger numbers of young adult acute leukaemia patients or at teaching hospitals. It is impossible to be certain why there should have been higher early mortality among AML patients at non-teaching hospitals or smaller centres. Severely ill patients could well be less likely to be referred to a tertiary centre, but differences in supportive care cannot be ruled out.

It is perhaps not surprising that we should have found less evidence of survival variations by place of treatment than in earlier studies of childhood leukaemia. The childhood studies would have included virtually all children treated by paediatric specialists, whereas patients aged 15–29 represent 42% of persons aged 15–64 with ALL and only 15% of those with AML (Cartwright et al, 1997). Thus, even hospitals with only one new patient per year with acute leukaemia aged 15–29 could well have seen 4–5 patients per year aged 15–64. Most patients were treated according to a standard MRC or other protocol (Benjamin et al, 1997), consistent with the absence of any variation in survival with hospital category even among non-MRC patients. Many were not actually entered in MRC trials, inevitably lessening the ability of these trials to answer rapidly the questions that they were set up to investigate.

The entry rate to the trials was lower at non-teaching hospitals and those with smaller numbers of leukaemia patients, especially for ALL. A very large number of hospitals participated in AML10, with an average entry rate of fewer than two patients per centre per year (Hann et al, 1997), but this did not prevent the attainment of exceptionally high survival rates. We also found no difference in survival by place of treatment for trial patients. Thus, while greater centralization of treatment might result in higher recruitment to trials, this could also be achieved by improving the entry rate from smaller centres without compromising survival. The reasons for lower participation rates from smaller centres require further study.

© Cancer Research Campaign 1999

One possible reason for the poor survival in an earlier period of children who received their treatment for ALL at a centre with few such patients *and* outside the MRC trials may be that these were the children who did not benefit from the collective experience within a specialist centre or a cooperative trials group. For adults with acute leukaemia, apart from the fact that most of them are treated according to accepted protocols, the growth of collaborative regional haematology groups provides the necessary pooling of experience to bring about uniformity of care (Charlton et al, 1997). Collaboration also facilitates the collection of high-quality, unbiased data, making possible studies such as the one reported here. This pattern is spreading to more areas of the country; registers of leukaemia patients in the West Midlands and former South-East Thames regions have been established, though too recently for inclusion in this study.

Treatment of adolescents in specialist oncology units dedicated to this age group has been advocated on two main grounds, medical and social, the medical case being founded on higher survival rates. In a survey of haematology departments in our study regions, we found hardly any units reserved for adolescents or young adults and therefore could not address directly the question of survival rates in adolescent units (Benjamin et al, 1997). However, our results provide no evidence of a survival advantage associated with the centralized treatment of acute leukaemias in this age group. The social case is that young people would prefer to be treated in a different environment from other, predominantly elderly cancer patients. Alternatively, adolescents and young adults may prefer to be treated near to where their friends live, and the necessity of travelling long distances and being resident for long periods in tertiary referral centres could result in depression and non-compliance with treatment. The present study could not provide any evidence on this point. However, while moves are afoot to provide more dedicated units for the treatment of patients with solid tumours in the age group discussed here it must remain in doubt whether such developments would have any impact on survival from leukaemia.

### ACKNOWLEDGEMENTS

We are grateful to the many consultants who provided information on their patients to the leukaemia registries and the Data Collection Study. We thank the National Health Service Central Register for flagging of survivors and provision of death certificates. We thank Mr M Gilbert and Mr MJ Loach for help with computing, Rachel Clack for preparing the data on MRC trials in the CTSU database, Mrs EM Roberts for secretarial assistance, and Ms A Berry and Miss A Sabin for clerical assistance.

This study was funded by a grant from the Leukaemia Research Fund, which also supports the LRF Data Collection Study. The Childhood Cancer Research Group is supported by the Department of Health; the views expressed in this publication are not necessarily those of the Department of Health. Dr PRA Taylor, data manager for the Northern Regional Haematology Group, has been supported by the Tyneside Leukaemia Research Fund and the Northern Regional NHS Research and Development Fund. Collection of data in the North-West Region was funded by the Lancaster and District Leukaemia Research Fund. The Welsh Leukaemia Register is funded by the Leukaemia Research Appeal for Wales. The Oxford Region Leukaemia Register is supported by the Haematology Research and Development Fund, Northampton General Hospital. The Mersey Regional Leukaemia Register is supported by locally collected research funds at the Countess of Chester Hospital. The Clinical Trial Service Unit is funded by the Medical Research Council for the leukaemia trials.

### REFERENCES

Badrinath P, Day NE and Stockton D (1997) Population-based survival trends for leukaemia in East Anglia, United Kingdom. *J Publ Health Med* **19**: 403–407

Benjamin S, Cartwright RA, Clough JV, Gorst DW, Proctor S, Ross JRY, Stiller C, Wheatley K and Whittaker JA (1997) Haematologists' approaches to the management of young adults with acute leukaemia (abstract). *Br J Haematol* **97** (suppl. 1): 56

Cartwright RA, McNally RJQ, Rowland DJ, Thomas J, Gilman E, Gorst D, Morgan G, Prentice A, Staines A and Stiller C (1997) *The Descriptive Epidemiology of Leukaemia and Related Conditions in Parts of the United Kingdom 1984–93*. Leukaemia Research Fund: London

Charlton BG, Taylor PRA and Proctor SJ (1997) The PACE (population-adjusted clinical epidemiology) strategy: a new approach to multi-centre clinical research. *Q J Med* **90**: 147–151

Chessells JM, Hall E, Prentice HG, Durrant J, Bailey CC and Richards SM (1998) The impact of age on outcome in lymphoblastic leukaemia; MRC UKALL X and XA compared: a report from the MRC Paediatric and Adult Working Parties. *Leukemia* **12**: 463–473

Durrant IJ and Richards SM (1993) Results of Medical Research Council trial UKALL IX in acute lymphoblastic leukaemia in adults; report from the Medical Research Council Working Party on adult leukaemia. *Br J Haematol* **85**: 84–92

Durrant IJ, Prentice HG and Richards SM (1997) Intensification of treatment for adults with acute lymphoblastic leukaemia: results of U.K. Medical Research Council randomized trial UKALL XA. *Br J Haematol* **99**: 84–92

Evensen SA, Brinch L, Tjonnford G, Stavem P and Wisloff F (1994) Estimated 8-year survival of more than 40% in a population-based study of 79 adult patients with acute lymphoblastic leukaemia. *Br J Haematol* **88**: 88–93

Expert Advisory Group on Cancer (1995) *A Policy Framework for Commissioning Cancer Services* (Calman and Hine Report). Department of Health: London

Goldstone AH, Lazarus HM, Franklin IM, Richards S, Anderson JW and Rowe JM (1997) Bone marrow transplantation versus chemotherapy in acute lymphoblastic leukaemia. Joint trial of the Medical Research Council (UKALL-12) with the United States Eastern Cooperative Oncology Group E2993. *Haematol Blood Trans* **38**: 700–703.

Gorst DW and Johnson KW (1992) Survival in adult leukaemia. *Clin Lab Haematol* **14**: 99–108

Hann IM, Stevens RF, Goldstone AH, Rees JKH, Wheatley K, Gray RG and Burnett AK (1997) Randomised comparison of DAT versus ADE as induction chemotherapy in children and younger adults with acute myeloid leukaemia. Results of the Medical Research Council's 10th AML trial (MRC AML10). *Blood* **89**: 2311–2318

Haward RA (1995) Establishing cancer units. *Br J Cancer* **72**: 531–534

Kerr DJ, Griffiths R and Edwards B (1996) Delivering cancer care: a model from the West Midlands. *Br J Cancer* **74**: 667–669

Marcus RE, Catovsky D, Prentice HG, Newland AC, Chessells JM, Stevens RF, Hann IM, Goldman JM, Hoffbrand AV and Galton DA (1987) Intensive induction and consolidation chemotherapy for adults and children with acute myeloid leukaemia (AML). Joint AML trial 1982–85. *Haematol Blood Trans* **30**: 346–351

Nachman J, Sather HN, Buckley JD, Gaynon PS, Steinherz PG, Tubergen DG, Lampkin BC and Hammond GD (1993) Young adults 16–21 years of age at diagnosis entered on Childrens Cancer Group acute lymphoblastic leukaemia and acute myeloblastic leukaemia protocols. Results of treatment. *Cancer* **71**: 3377–3385.

Proctor SJ (1994) Acute lymphoblastic leukaemia in adults: the case for a strategic shift in study approach. *Br J Haematol* **88**: 229–233

Proctor SJ, Taylor P, Thompson RB, Finney R, Reid MM, Hamilton PJ, Saunders P, Fail B, Dickinson A, Paul B, Qureshi M, Tinegate H, Lennard A, Stainsby D, Goff D, Kay L, Cartner R, Mamood A, Bird T, Condie P, Collins A, Abela M, Renwick L, Walker W and Evans RGB (1985) Acute lymphoblastic leukaemia in adults in the Northern Region of England – a study of 75 cases. *Q J Med* **57**: 761–774

Proctor SJ, Taylor PRA, Stark A, Carey PJ, Bown N, Hamilton PJ and Reid MM (1995) Evaluation of the impact of allogeneic transplant in first remission on an unselected population of patients with acute myeloid leukaemia aged 15–55 years. *Leukemia* **9**: 1246–1251

 *© Cancer Research Campaign 1999*

Rees JKH, Gray RG and Wheatley K (1996) Dose intensification in acute myeloid leukaemia: greater effectiveness at lower cost. Principal report of the Medical Research Council's AML 9 study. *Br J Haematol* **94**: 89–98

Secker-Walker LM, Prentice HG, Durrant J, Richards S, Hall E and Harrison G (1997) Cytogenetics adds independent prognostic information in adults with acute lymphoblastic leukaemia on MRC trial UKALL XA. *Br J Haematol* **96**: 601–610

Selby P, Gillis C and Haward R (1996) Benefits from specialised cancer care. *Lancet* **348**: 313–318

Souhami RL, Whelan J, McCarthy JF and Kilby A (1996) Benefits and problems of an adolescent oncology unit. In *Cancer and the Adolescent*, Selby P and Bailey C (eds), pp. 276–283. BMJ: London

Stiller CA (1994*a*) Population based survival rates for childhood cancer in Britain, 1980–91. *BMJ* **309**: 1612–1616

Stiller CA (1994*b*) Centralised treatment, entry to trials and survival. *Br J Cancer* **70**: 352–362

Stiller CA and Draper GJ (1989) Treatment centre size, entry to trials and survival in acute lymphoblastic leukaemia. *Arch Dis Child* **64**: 657–661

Stiller CA and Eatock EM (1994) Survival from acute non-lymphocytic leukaemia, 1971–88: a population-based study. *Arch Dis Child* **70**: 219–223

Taylor I (1996) Problems of site specific cancer specialisation. *BMJ* **313**: 1213

Warrell RP, Frankel SR, Miller WH, Scheinberg DA, Itri LM, Hittelman WN, Vyms R, Andreeff M, Tafuri A, Jakubowski A, Gabrilove J, Gordon MS and Dmitrovsky E (1991) Differentiation therapy of acute promyelocytic leukaemia with tretinoin (all-trans-retinoic acid). *N Engl J Med* **324**: 1385–1393

Youngson JHAM, Jones JM, Chang JG, Harris M and Banergee SS (1995) Treatment and survival of lymphoid malignancy in the north-west of England: a population-based study. *Br J Cancer* **72**: 757–765

© Cancer Research Campaign 1999
